# The first wave of pandemic influenza (H1N1) 2009 in Germany: From initiation to acceleration

**DOI:** 10.1186/1471-2334-10-155

**Published:** 2010-06-07

**Authors:** Gabriele Poggensee, Andreas Gilsdorf, Silke Buda, Tim Eckmanns, Hermann Claus, Doris Altmann, Gérard Krause, Walter Haas

**Affiliations:** 1 Department for Infectious Disease Epidemiology, Robert Koch Institute, DGZ-Ring 1, 13086 Berlin, Germany

## Abstract

**Background:**

The first imported case of pandemic influenza (H1N1) 2009 in Germany was confirmed in April 2009. However, the first wave with measurable burden of disease started only in October 2009. The basic epidemiological and clinical characteristics of the pandemic were analysed in order to understand the course of the pandemic in Germany.

**Methods:**

The analysis was based on data from the case-based, mandatory German surveillance system for infectious diseases. Cases notified between 27 April and 11 November 2009 and fulfilling the case definition were included in the study.

**Results:**

Two time periods with distinct epidemiologic characteristics could be determined: 23,789 cases (44.1%) occurred during the initiation period (IP, week 18 to 41), and 30,179 (55.9%) during the acceleration period (AP, week 42 to 45). During IP, coinciding with school summer holidays, 61.1% of cases were travel-related and one death occurred. Strict containment efforts were performed until week 32. During AP the majority of cases (94.3%) was autochthonous, 12 deaths were reported. The main affected age group shifted from 15 to 19 years in IP to 10 to 14 years in AP (median age 19 versus 15 years; p < 0.001). The proportion of cases with underlying medical conditions increased from 4.7% to 6.9% (p < 0.001). Irrespective of the period, these cases were more likely to be hospitalised (OR = 3.6 [95% CI: 3.1; 4.3]) and to develop pneumonia (OR = 8.1 [95% CI: 6.1; 10.7]). Furthermore, young children (0 to 2 years) (OR = 2.8 [95% CI: 1.5; 5.2]) and persons with influenza-like illness (ILI, OR = 1.4 [95% CI: 1.0; 2.1]) had a higher risk to develop pneumonia compared to other age groups and individuals without ILI.

**Conclusion:**

The epidemiological differences we could show between summer and autumn 2009 might have been influenced by the school summer holidays and containment efforts. The spread of disease did not result in change of risk groups or severity. Our results show that analyses of case-based information can advise future public health measures.

## Background

Only a few days after the World Health Organization announced on 24 April 2009 a Public Health Emergency of International Concern caused by a new influenza virus variant, the first imported case of pandemic influenza (H1N1) 2009 was confirmed in Germany [1]. International travel facilitated the geographical spread from the initial foci of infection in Mexico and the United States to many countries throughout the world, seeding urban centres with a high intensity of transmission before wider geographical spread within countries occurred [2]. In summer 2009, most countries reported increasing case numbers, however, differences in patterns and intensity of transmission ranging from sustained human-to-human transmission in some areas to local transmission in others were observed. While on average 50% of the cases in Europe were travel-related during the summer months, noticeable geographical differences were seen. In July 2009, in the UK a sharp increase in the proportion of locally acquired infections was observed [3]. At the same time, in Germany the proportion of cases associated with travel to European countries continued to rise, with the majority of infections in travellers returning from Spain - a major holiday destination [4]. In contrast to other European countries no influenza-associated deaths were reported until 25 September 2009. The first wave of the H1N1 pandemic with measurable disease burden at the population level in Germany started only in October 2009.

Based on the surveillance data we analysed the basic epidemiological and clinical characteristics of pandemic influenza (H1N1) 2009 infection in order to understand the course of the pandemic in Germany. Furthermore we investigated factors associated with hospitalisation and the occurrence of pneumonia to provide new as well as verify existing evidence on risk groups for severe disease and resulting recommendations.

## Methods

The analyses were based upon data from two sources: The national surveillance system for notifiable infectious diseases and the sentinel surveillance system for acute respiratory diseases (ARI).

In 2001, the German national electronic surveillance system (SurvNet@rki) was established. Case-based information is transmitted electronically by the local health authorities via the state health authorities to the Robert Koch Institute, which is the national public health institute (RKI) [5]. Transmitted data sets include information on age, sex, date of symptom onset, hospitalisation, and country of infection, amongst others.

Cases notified between 27 April 2009 (week 18) and 11 November 2009 (week 45) which fulfilled the following case definition were included in the analyses:

• Any case with detection of nucleic acid of pandemic influenza (H1N1) 2009 by real-time PCR [6], regardless of clinical presentation (laboratory-confirmed case),

• any case with fever and acute respiratory symptoms or death from unexplained acute respiratory illness with contact to a laboratory-confirmed case (epidemiological linked case).

Influenza-like illness is defined as acute onset of respiratory symptoms, and fever, and cough, and headache or musculo-skeletal pain. Risk factors for severe courses are defined as chronic diseases (cardio-vascular disease, respiratory disease, immunosuppression, diabetes, other chronic diseases), obesity (body mass index > 30), and pregnancy. This additional information was gathered for each case using a defined free-text format available in SurvNet@rki starting in week 18 and expanded to retrieve more detailed information in weeks 26 and 43 (see annex).

The burden of disease is measured by syndromic sentinel surveillance. The sentinel surveillance system of acute respiratory illness (ARI; defined as acute pharyngitis, bronchitis or pneumonia with or without fever) was established in Germany in 1992, since summer 2006 a year-round sentinel surveillance of influenza was implemented. Since week 16/2009 the sentinel surveillance is exclusively performed by the sentinel physicians and the RKI. Data from approx. 400 (summer) to 650 (winter) actively reporting sentinel sites are transmitted weekly to the Robert Koch Institute. An index ("Practice index"; PI) for each participating sentinel site (surgery/practice) is calculated weekly by the "Arbeitsgemeinschaft Influenza" (http://influenza.rki.de). The PI is defined as the relative deviation of observed consultations for ARI divided by all consultations in the same week and set into relation to the background value of this ratio in weeks without influenza virus circulation. Background ARI activity during winter is set at the threshold PI of 115.

Age was calculated based on the notified year and month of birth. The age group of school-aged children was defined as 5 to 14 years. Categorical variables are presented as percentages with interquartile ranges and continuous variables are presented with interquartile range where appropriate. Bivariate analysis was done to compare two groups using the chi-square test for categorical and the Mann-Whitney test for continuous variables. Odds were calculated including 95% confidence intervals. Unconditional logistic regression models were performed including all variables of interest to investigate the association of hospitalisation and pneumonia with independent variables. Analyses were performed using PASW Statistics 17.0 (SPSS Inc, Chicago, USA).

As one of the possible factors with impact on the course of the pandemic the timing of the school holidays was analysed. In Germany the school vacation times differ between the 16 Federal States. We calculated the vacation density for the observation period and defined it as the mean proportion of the population per week living in States with on-going school holidays (http://www.schulferien.org/inverser_Ferienkalender/ferientermine_2009_invers.html).

## Results

### General epidemiological features

As of 11 November 2009 a total of 53,968 cases and 13 deaths was reported from week 18 through week 45 to the RKI. The most frequent reported symptoms were fever (80.7%) and cough (77.8%). Acute onset of disease was reported in 22.4% of the cases.

**Figure 1:**
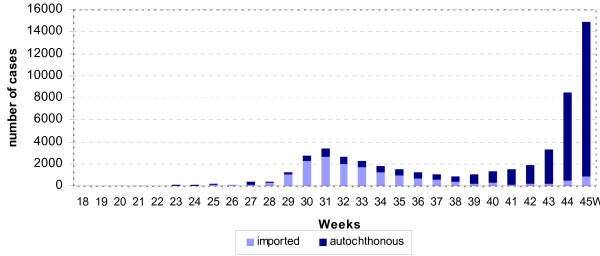
**Cases of pandemic influenza (H1N1) 2009 virus infection in Germany by source of origin by week as of 11 November 2009**. (n = 53,968).

**Figure 2:**
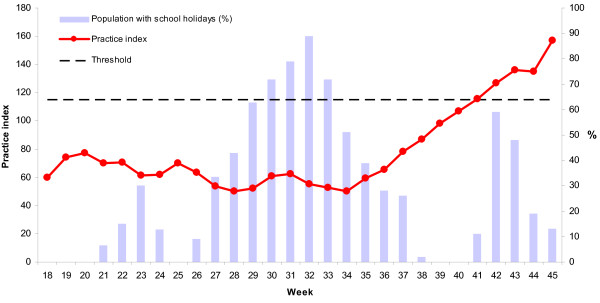
**Activity of acute respiratory illness and school holiday density between week 18 and 45, Germany 2009**. Weekly data of Practice index in Germany. Threshold >115. (dotted line) Arbeitsgemeinschaft Influenza, Robert Koch Institute. Light blue columns indicate the vacation density for each week.

Weeks 18 through 28 were characterised by low numbers of reported cases. From week 29 to 35 an increased number of cases was reported with a peak of more than 3,500 cases in week 31. The peak was followed by a lower plateau with 850 to 1,500 newly reported cases per week (week 36 to 41). From week 42 onwards the number of reported cases started to increase steeply forming the first wave of autochthonous transmission of pandemic influenza (H1N1) 2009 in Germany (figure 1). During the summer no increased influenza activity was detected by the syndromic sentinel surveillance, however, from week 42 onwards the nationwide ARI activity was for the first time above the baseline activity threshold with pronounced increased activity in the southern part of Germany, especially in Bavaria (figure 2).

**Figure 3:**
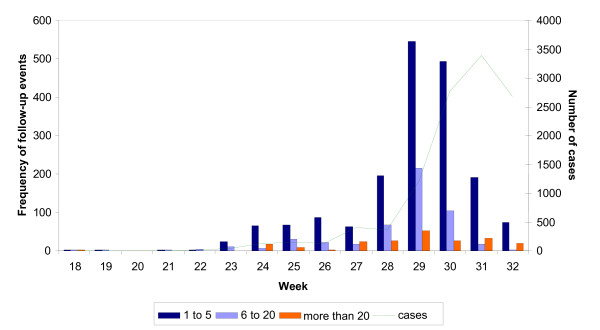
**Number of trace back events by number of contacts traced per case with pandemic influenza (H1N1) 2009 infection in Germany, week 18 to 32 in 2009**. The green line represents the number of notified pandemic influenza (H1N1) 2009 cases; the bars present the number of contact persons followed-up per case of pandemic influenza A/c.

From week 18 to 32 information on contact tracing was available for 2,896 (26%) of 11,283 notified cases (figure 3). For these cases 21,717 contact persons were reported. In average (median), 3 contact persons per case were identified and followed up (lower and upper quartile: 2, 6; range: 1 person to 330 persons), no contact persons were traced for 133 pandemic influenza cases (4.6%). For 2,388 follow-up events information was available on whether antiviral prophylaxis has been given to the contact persons. Between week 18 and 23 antiviral prophylaxis was given to 71.0% to 100% of the contact persons dropping to 10.3% in week 32.

We defined an initiation and acceleration period of the pandemic in Germany based on the results of the syndromic sentinel surveillance and routine surveillance:

Initiation period with cases diagnosed between week 18 and 41 and acceleration period with cases diagnosed in weeks 42 to 45. From week 42 onwards the number of notified cases started to rapidly increase for the second time after the smaller peak in week 31. Furthermore, in week 42 the Practice Index increased above the background winter activity of acute respiratory infection (threshold: PI >115).

### Comparison of periods

In table 1 the comparison of cases of the initiation period versus cases of the acceleration period is shown: Out of the 53,968 cases reported to the RKI as of 11 November 2009, 23,789 cases (44.1%) occurred during the initiation period and 30,179 cases (55.9%) during the acceleration period.

**Table 1:** Characteristics of cases of pandemic influenza reported in the initiation period (week 18 to week 41, 2009) and acceleration period (week 42 to week 46, 2009)

	Number of cases with information	Initiation period (%)^a^	Acceleration period (%)^a^
**Total**	53,968	23,789 (44.1)	30,179 (55.9)
			
**Age groups**	53,930	23,783	30,147
Median [quartiles]		19 [16,26]^#^	15 [10,26]^#^
0-4 years	2,076	516 (2.2)	1,560 (5.2)
5-14 years	16,989	3,794 (16.0)	13,195 (43.8)
15-24 years	20,295	12,854 (54.0)	7,441 (24.7)
25-59 years	13,975	6,398 (26.9)	7,577 (25.1)
≥ 60 years	595	221 (0.9)	374 (1.2)
			
**Origin of infection**	53,757	23,714	30,043
Autochthonous cases	37,573	9,235 (38.9)	28,338 (94.3)
			
**Risk factors**	39,748	17,579	22,169
All cases		820 (4.7)*	1,539 (6.9)*
Autochthonous cases	27,486	411/6,868 (6.0)**	1,458/20,618 (7.1)**
Imported cases	12,140	405/10,644 (3.8)**	77/1,476 (5.2)**
			
Risk factors			
Chronic diseases			
Respiratory disease	1,080	372 (2.1)	708 (3.2)
Cardio-vascular disease	264	107 (0.6)	157 (0.7)
Diabetes	173	63 (0.4)	110 (0.5)
Obesity^§^	159	71 (0.4)	88 (0.4)
Immunosuppression	112	40 (0.2)	72 (0.3)
Other chronic diseases	458	127 (0.7)	331 (1.5)
Pregnancy	113	40 (0.2)	73 (0.3)
			
Age groups	39,748	17,533	22,043
0-4 years	1,474	17/367 (4.6)	53/1.107 (4.8)
5-14 years	12,351	101/2,831 (3.6)	571/9,520 (6.0)
15-24 years	15,107	314/9,510 (3.3)	291/5,597 (5.2)
25-59 years	10,368	350/4,711 (7.4)	560/5,657 (9.9)
≥ 60 years	429	38/157 (24.2)	63/272 (23.2)
			
**Symptoms**	53,968	23,789	30,179
ILI		2,318 (9.7)	2,924 (9.7)
			
**Diagnosis**			
Time interval between onset of symptoms and diagnosis	7.253	3 days [2, 5]^#^	2 days [1,4]^#^
			
**Antiviral treatment initiated**	35,160	15,941	19,219
All cases		4,671 (29.3)	4.491 (24.7)
Cases with underlying chronic disease	2,092	319/743 (42.9)	598/1,278 (46.8)
Cases without risk factors	33,139	4,352/15,198 (28.6)	3,893/17,941 (21.7)
Pregnancy	88	6/34 (17.6)	13/54 (24.1)
Time interval between symptom onset and start of therapy		2 days [1,3]^#^	1 day [1,2]^#^
			
**Pneumonia**	40,729	18,090	22,639
All cases with pneumonia	275	93 (0.5)	182 (0.8)
Antiviral treatment initiated	219	33/77 (42.9)	56/142 (39.4)
Cases with underlying chronic disease	76	19/764 (2.5)	57/1,410 (4.0)
Cases without risk factors	176	66/16,303 (0.4)	110/20,192 (0.5)
Pregnancy	99	1/37 (2.7)	1/62(1.6)
			
**Hospitalisation**	52,485	23,665	28,820
All cases hospitalised	2,084	1,117 (4.7)*	967 (3.4)*
Age (years)		19 [16,26]^#^	15 [11,26]^#^
Cases with risk factors	2,342	102/814 (12.5)	182/1,528 (11.9)
Age (years)		23 [16,41] #	17 [11, 39] #
Cases without risk factors	37,187	618/16,711 (3.7)*	597/20.476 (2.9)*
Age (years)		19 [16,25]^#^	15 [10,25]^#^

^# ^Lower/upper quartile

^§ ^Obesity requiring treatment or body mass index > 30

^a ^The percentages in brackets refer to the number of cases with available information regarding the variable. In each cell the number printed in bold is used as denominators in a cell. If another denominator is used, it is given in the table.

* Comparison initiation versus acceleration period: p < 0.001

** Comparison initiation versus acceleration period: p < 0.01

During the initiation period, 61.1% of cases were travel-related, whereas during the acceleration period 94.3% were autochthonous (table 1). The distribution over time is shown in figure 1.

**Figure 4:**
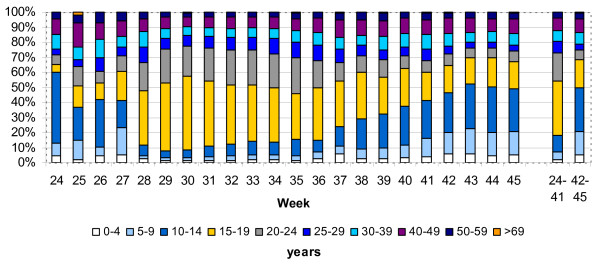
**Age distribution of cases with pandemic influenza (H1N1) 2009 infection in Germany from week 24 to week 45 in 2009; age distribution in weeks 24 to 41 (initiation period) and in weeks 42 to 45 (acceleration period)**.

Figure 4 shows the age distribution of cases over the weeks. In the initiation period the age group 15 to 24 years constituted 54% of all cases (n = 12,854) dropping to 24.7% (n = 7.441) in the acceleration period. The proportion of school-aged children in the age group 5 to 14 years increased from 16% (n = 3,794) in the initiation period to 43.8% (n = 13,195) in the acceleration period. The median age decreased significantly from 19 years in the initiation period to 15 years in the acceleration period (p < 0.001; table 1).

On average, 29% of the German population lived in States with school holidays during the observation period between weeks 18 and 45. Vacation density peaks in the time period between weeks 29 and 34 reflecting the main school holidays during summer months in Germany and coinciding with the peak of reported cases during the initiation period. No marked difference in the mean weekly vacation density between the two time periods was found (initiation period: 29%; acceleration period: 30%). However, after week 37 the vacation density dropped to 0% for two continuous weeks (39 and 40) just prior to the rise above the threshold of the syndromic surveillance (figure 2).

The proportion of cases where the reported symptoms were in accordance with the case definition for influenza-like illness (ILI) did not differ between the two periods and was as low as 9.7% (Table 1).

In table 1, information is given on risk factors of cases (chronic diseases, obesity, pregnancy) during the two periods. Overall the proportion of cases with reported risk factors was significantly higher in the acceleration period compared to the initiation period (6.9% vs. 4.7%; p < 0.001). The proportion of reported cases with risk factors increased in the acceleration period for all age groups except for cases older than 60 years. In both periods the proportion of cases with risk factors was significantly higher in patients who acquired the infection in Germany (initiation period: 5.7% versus 3.6%; p < 0.004; acceleration period: 6.6% versus 4.8%: p < 0.0001).

The time interval between onset of symptoms and the time of diagnosis decreased from the initiation to the acceleration period from 3 days to 2 days and the time between onset of symptoms and initiation of treatment from 2 days to 1 day (table 1). The proportion of cases with initiated antiviral treatment dropped from 29.3% in the initiation period to 24.7% in the acceleration period, while it increased for cases with and decreased for cases without underlying conditions.

During the initiation period a higher proportion of cases was hospitalised (4.7% versus 3.4%) and the median age of hospitalised patients was 19 years compared to 15 years in the acceleration period.

The analysis of factors associated with hospitalisation and development of pneumonia (table 2) showed that pneumonia was highly associated with being hospitalised (OR = 25.5 [95% CI: 20.0; 32.5]). Among risk factors for hospitalisation, immunosuppression (OR = 10.8 [95% CI: 7.1; 16.3]) and pregnancy (OR = 8.6 [95% CI: 5.5; 13.5]) were also highly associated with hospitalisation. In the univariate analysis highest risks of developing pneumonia were seen in cases with immunosuppression (OR = 14.9 [95% CI: 6.9; 32.7]) and obesity (OR = 13.8 [95% CI: 6.9; 27.5]). Having any risk factor was associated with a 7.6-higher risk of developing pneumonia [95% CI: 5.7; 9.8].

**Table 2:** Factors influencing hospitalisation of individuals affected by pandemic influenza (H1N1) 2009 and risk factors for the development of pneumonia.

Independent variables	n	Individuals hospitalised/with pneumonia (%)	Univariate analysis OR (95%CI)	p-value	Multivariate analysis OR (95%CI)	p- value
**Hospitalisation**						
Pneumonia	275	130 (47.3)	25.5 (20.0;32.5)	<0.001	18.2 (13.6;23.9)	<0.001
No pneumonia reported	40,236	1,366 (3.4)	reference			
						
Risk factors present	2,342	284 (12.1)	4.6 (3.6;4.7)	<0.001	3.6 (3.1;4.3)	<0.001
No risk factor reported	37,187	1,215 (3.3)	Reference			
						
Chronic disease	2,250	261 (11.6)	3.8 (3.3;4.4)	<0.001		
No chronic disease reported	37,298	1,240 (3.3)	Reference			
Immunosuppression	112	30 (26.8)	10.8 (7.1;16.3)	<0.001		
No risk factor reported	37,187	1,215 (3.3)	reference			
Respiratory disease	1,072	87 (8.1)	2,6 (2,1; 3,3)	<0.001		
No risk factor reported	37,187	1,215 (3.3)	reference			
Obesity*	159	19 (11.9)	4.0 (2,5; 6,5)	<0.001		
No risk factor reported	37,187	1,215 (3.3)	reference			
Cardio-vascular disease	260	34 (13.1)	4.5 (3.1; 6.4)	<0.001		
No risk factor reported	37,187	1,215 (3.3)	reference			
Diabetes	172	19 (11.0)	3.7 (2.3; 5.9)	<0.001		
No risk factor reported	37,187	1,215 (3.3)	reference			
Other chronic disease	70	70 (15.4)	5.4 (4.1; 7.0)	<0.001		
No risk factor reported	37,187	1,215 (3.3)	reference			
Pregnancy	106	25 (25.6)	9.9 (6.1;16.0)	<0.001		
No pregnancy reported	16,957	527 (3.1)	reference			
Age						
0 - 2 years	920	107 (11.6)	3.3 (2.7;4.1)	<0.001	2.9 (2.2;3.8)	<0.001
All other age groups	51,563	1,977 (3.8)	reference			
						
3 - 14 years	17,486	509 (2.9)	0,6 (0.5;0.7)	<0.001	0.7 (0.6;0.8)	<0.001
All other age groups	34,997	1,575 (4.5)	reference			
						
Male	27,345	1,114 (4.1)	1.1 (0.9;1.2)	= 0.069	1.1 (0.9;1.2)	= 0.224
Female	24,628	927 (3.8)	reference			
						
Acceleration period	28,820	967 (3.4)	0.7 (0.6; 0.8)	<0.001	0.8 (0.7;0.9)	= 0.020
Initiation period	23,663	1,117 (4.7)	reference			
**Pneumonia**						
Risk factors	2,162	76 (3.5)	7.4 (5.7;9.8)	<0.001	8.1 (6.1;10.7)	<0.001
No risk factor reported	36,305	177 (0.5)	reference			
Chronic disease	2,082	75 (3.6)	7.6 (5.8;9.9)	<0.001		
No chronic disease reported	36,402	179 (0.5)	reference			
Immunosuppression	103	7 (6.8)	14.9 (6.9;32.7)	<0.001		
No risk factor reported	36,305	177 (0.5)	reference			
Respiratory disease	994	34 (3.4)	7.2 (4.9;10.5)	<0.001		
No risk factor reported	36,305	177 (0.5)	reference			
Obesity*	143	9 (6.3)	13.8 (6.9;27.5)	<0.001		
No risk factor reported	36,305	177 (0.5)	reference			
Cardio-vascular disease	243	5 (2.1)	4.3 (1.7;10.4)	= 0.002		
No risk factor reported	36,305	177 (0.5)	reference			
Diabetes	157	1 (0.6)	1.3 (0.2;9.4)	= 0.790		
No risk factor reported	36,305	177 (0.5)	reference			
Other chronic disease	425	18 (4.2)	9.1 (5.5;14.8)	<0.001		
No risk factor reported	36,305	177 (0.5)	reference			
Pregnancy	95	2 (2.1)	4.6 (1.1;19.0)	= 0.074		
No pregnancy reported	17,194	80 (0.5)	reference			
						
Age						
0 - 2 years	712	15 (2.1)	3.3 (1.9;5.5)	<0.001	2,8 (1.5;5.2)	<0.001
All other age groups	40,019	261 (0.7)	reference			
3 - 14 years	13,453	92(0.7)	1.1 (0.8;1.3)	= 0.914		
All Other age groups	27,278	184 (0.7)	reference			
						
Male	21,158	146 (0.7)	1.1 (0.7;1.4)	= 0.413	1.1 (0.8;1.4)	= 0.545
Female	19,228	120 (0.6)	reference			
						
Acceleration period	22,640	182 (0.8)	1.6 (1.2;2.0)	= 0.001	1.3 (1.0;1.7)	= 0.038
Initiation period	18,081	94 (0.5)	reference			
						
ILI**	4,442	39 (0.9)	1.3 (1.0;1.8)	= 0.090	1.4 (1.0;2.1)	= 0.032
No ILI	36,289	237 (0.7)	reference			
						
Fever (>38.5°C)	34,905	258 (0.7)	1.9 (1.2;3.1)	= 0.007	2.0 (1.2;3.5)	= 0.013
No fever reported	4,826	18 (0.4)	reference			

* Obesity requiring treatment or body mass index > 30

** ILI: acute onset of symptoms and fever and cough and headache or musculoskeletal pain

In the multivariate analyses controlling for the period of the pandemic, patients with risk factors and children aged 0 to 2 years were more likely to be hospitalised. Furthermore, these patient groups and additionally individuals who had ILI or fever were more likely to develop pneumonia.

## Discussion

The analysis of the first 50,000 cases of pandemic influenza (H1N1) 2009 in Germany showed a first increase of cases in early summer comparable to the epidemiological situation in other European countries in week 31 and 32 [7,8], but the first wave with autochthonous cases occurred later than e.g. in the United Kingdom, which saw a considerable increase of cases already as early as week 28 [9]. In Germany, despite a relevant number of cases per week in summer, the peak levelled off and the first wave only started in week 42. With descriptive analysis of surveillance data from mandatory notification and syndromic surveillance of acute respiratory illness we show that the epidemiologic characteristics differed markedly between the summer months and the fall, allowing definition of two distinct time periods.

One of the most obvious differences observed was the proportion of imported cases in both periods. Over half of the cases were imported in the initiation period, while only 5% in the acceleration period were travel-related. During the summer, compared to other European countries a much lower in-country transmission occurred in Germany. ECDC reported in beginning of August that only 29% of reported cases in Europe were travel-related [10].

A very clear age shift between the two periods was noticed. In the initiation period Germany had a very high proportion of cases in the 15 to 24 years age group, in the later period there was a strong shift to the younger 5 to 14 years age groups. A large proportion of cases in the summer peak were high school graduates and young adults, who got infected while on summer travels. Behavioural factors of the young adults during the holidays (such as close contacts with peers, partying) might have had an influence on the transmission within this age group [11]. It is known, that school children play an important role in the transmission of influenza [12-14]. But during the summer peak, pandemic influenza did hardly spread within this group in Germany. This might be explained by the timing of the summer school holidays which started between weeks 27 and week 31 in the different Federal States (Figure 2). Since mid October, after the autumn holidays the school-aged children started to be more affected.

Another factor that could have contributed to a reduced transmission in the summer was the relatively strict contact tracing efforts done by the local public health authorities [15]. Contact tracing resulted mainly in recommendations to cases and household contacts including personal hygiene (e.g., hand washing, use of face masks, and segregation within the household) and prophylactic antiviral treatment. It has been described that non-pharmaceutical interventions can reduce the transmission of influenza within households if implemented within 36 hours of the index patient's symptom onset [16]. Individual case management efforts dwindled in most local public health authorities at the peak of the initiation period (week 32), as the number of cases became too high to be able to continue contact tracing. More research is needed to assess to what extent public health measures and school holidays had an impact on the course of the pandemic in the initiation period.

The proportion of cases with risk factors increased significantly from initiation period to acceleration period. As the recommendation changed in August 2009 to only diagnose and treat persons with higher risks for developing severe disease, it was anticipated, that the reported number of cases without underlying conditions would drop. Furthermore, the fact that infection was associated with travel in the beginning of the influenza pandemic might have played a role ("healthy travellers"). It might be assumed that people with health problems travel less, and had therefore less chance of infection in the initiation period. It is conceivable, that the change in the proportion of cases with underlying conditions was more related to these factors than to a change of the pathogenicity of the virus, which so far did not occur [17].

The decrease in hospitalisation rates might be explained by physicians' better knowledge of the disease. In the beginning many affected individuals were hospitalised, either out of anxiety or for infection control reasons [1]. Early admission to hospital at the beginning of the pandemic due to uncertainty in relation to the clinical presentation and likely progression of disease has been described in other European Countries [7,18]. Furthermore, physicians were aware about the recommendations regarding the patient management. An increased time interval between onset of symptoms and diagnosis and/or treatment possibly due to higher numbers of cases in the acceleration period did not occur as there was no overloading of the health system in Germany. On the contrary, the opposite happened and - as recommended - treatment was initiated on average earlier during the acceleration period.

Other known factors might have also influenced the course of the pandemic in Germany, e.g. seasonality. In general, temperature and humidity can serve as proxy for seasonality and possibly have direct and indirect effects on the spread of disease. These parameters have been suggested to have an impact on the barrier function of the nasal mucosa, and on the other hand can influence behaviour (i.e., longer stay in confined spaces) thus increasing the risk of transmission [19,20]. As a proxy for this complex interaction of environmental factors, in week 42 a temperature drop was observed shortly before the start of the acceleration period in Germany. However, this observation requires a more detailed study in which regional geographic meteorological conditions and long-term trends are compared to the respective epidemiological dynamics of the pandemic in Europe.

In order to assess if the change in the dynamic of spread was also reflected by more severe disease, especially among defined priority groups, factors associated with hospitalisation and the development of pneumonia as proxies for severity were analysed. Unsurprisingly, pneumonia was highly associated with being hospitalised. As in other studies, immunosuppression and pregnancy were also highly associated with hospitalisation [7,21]. The univariate analysis of risk factors for developing pneumonia shows that all reported underlying conditions (except diabetes) were positively associated. It could be shown that obesity had a strong association, which has already been described in other studies [22,23]. However, the high association we found could also be seen as a proxy for underlying pulmonary or cardio-vascular diseases. The risk to have pneumonia was 8 times higher in people with risk factors compared to people without. The analyses draw a special focus on young children up to two years, who had nearly a three fold higher risk of being hospitalised and developing pneumonia compared to the other age groups. While the risk of hospitalisation was lower in the acceleration period; the risk of pneumonia among cases without underlying risk factors did not differ by time period.

Certain limitations of the study have to be kept in mind. The data collection with regard to additional information such as the underlying chronic diseases and pregnancy was performed by an additional free-text format. The format was limited in space, therefore only one risk factor (underlying chronic disease or obesity or pregnancy) could be reported and an analysis of the impact of multiple underlying health conditions was not possible. Bias might have been introduced by missing data on clinical parameters and therapy. Information on the risk factors were only collected from June 2009 onwards (see annex). Due to high case numbers in the acceleration period local health authorities might not have been able to collect the data due to limited human resources. Furthermore, clinical information and information on therapy were often not available. In the acceleration period, again due to limited resources, efforts to collect epidemiological data might have been biased toward more severe disease and hospitalised persons.

## Conclusions

The comparison of the epidemiological and clinical data of the pandemic influenza (H1N1) 2009 cases indicated that important differences could be observed between the initiation period during the summer months and the acceleration period with the beginning of the first wave. Different factors might have played a role: School holidays reducing the number of social contacts between school-aged children, the initial introduction of pandemic influenza in the group of adolescents and young adults, and seasonality. But also the strict containment strategy in the beginning of the pandemic could have contributed to the fact, that Germany experienced its first autochthonous wave much later than other European countries. More severe cases were seen in young children, in patients with initial influenza-like illness and individuals with underlying conditions. However, the spread of the disease did not result in a change of severity in Germany over time.

Our results show that the analyses of case-based data can help to anticipate changes of the epidemic and inform about mainly affected groups and therefore advice future public health measures.

## Competing interests

The authors declare that they have no competing interests.

## Authors' contributions

GP drafted the manuscript in consultation with AG, SB, TE, GK, and WH; GP conducted the analyses in consultation with AG, SB and WH; CH and DA were responsible for the data management; all authors read and approved the final manuscript. All members of the RKI-WGPI were involved in the response of the RKI to the pandemic in crisis management, and collection of the surveillance data.

## Appendix

### Public health strategies, enhanced surveillance and legal basis

According to the Infectious Disease Act (Infektionsschutzgesetz, IfSG) laboratories have to notify to the local health authorities the detection of influenza virus, this obligation includes new subtypes of influenza virus. On 30 April 2009, a legal act came into force, obliging physicians to report suspected cases, clinically diagnosed cases, confirmed cases, and deaths of pandemic influenza (H1N1) 2009.

Enhanced surveillance of pandemic influenza was implemented based on a surveillance case definition for pandemic influenza A/H1N1 differentiating between suspected, probable, and confirmed cases. With the evolution of the pandemic and the adaptation of the strategy from containment to protection and finally mitigation, the case definition was adopted three times (May 2009, August 2009, and October 2009).

Starting from 30 April 2009 (week 18) besides information gathered routinely for each notified case according to the IfSG, the following additional information was collected using a standardised free-text format: classification of cases (possible, probable, confirmed, discarded case), in-country transmission, number of contacts followed-up, antiviral prophylaxis of contacts. From 21 July 2009 onwards (week 29), the variables of the free-text were changed in order to collect more detailed data on the treatment of cases (start of therapy, antiviral drug used), risk factors (chronic diseases, pregnancy, obesity (body mass index > 30), presence of pneumonia, hospitalisation and source of infection. Starting on 20 October 2009 (week 43), information on vaccination against pandemic influenza was included.

After the detection of the first case, a public health response strategy of containment was established. Diagnosed cases were isolated (mainly home isolation). Close contacts of laboratory diagnosed cases of pandemic influenza (H1N1) 2009 (e.g., household members, medical care) were quarantined for 7 days and were asked to check the temperature twice daily and to report to the local health authority. Other contact persons were advised to check the temperature daily for 7 days, to avoid contact to vulnerable persons and to contact the local health authorities immediately at onset of symptoms. On 18 August 2009 (week 33) the public health response strategy changed, aiming now to protect vulnerable groups until the availability of a vaccine against pandemic influenza. Close contacts of cases working with vulnerable groups were requested to avoid contact with these groups; however, home quarantine was not requested any more. For other contacts only general information on pandemic influenza, health monitoring, and hygiene were given.

With the availability of the pandemic vaccine in October 2009 and the recommendations of the Standing Committee on Vaccination giving priority for the vaccination of medical staff and persons with risk factors, the protection of vulnerable groups by using contact management ceased to be a task of the local health authorities. At the same time vaccine prevention and early diagnosis and treatment of severe cases became the focus of the strategy to mitigate the consequences of the pandemic. On 2 November 2009 the notification act was changed, physicians have been only obliged to report deaths of persons if an infection with pandemic influenza (H1N1) 2009 was diagnosed in temporal connection with the death. End of October 2009 the vaccination against pandemic influenza started in all federal states.

## Pre-publication history

The pre-publication history for this paper can be accessed here:

http://www.biomedcentral.com/1471-2334/10/155/prepub
